# Response to: Comment on “Effects of a Pragmatic Home-Based Exercise Program Concurrent With Neoadjuvant Therapy on Physical Function of Patients With Pancreatic Cancer: The PancFit Randomized Clinical Trial”

**DOI:** 10.1097/AS9.0000000000000435

**Published:** 2024-05-14

**Authors:** An T. Ngo-Huang, Nathan H. Parker, Keri L. Schadler, Matthew H. G. Katz

**Affiliations:** * From the Department of Palliative, Rehabilitation, and Integrative Medicine, The University of Texas MD Anderson Cancer Center, Houston, TX; † Department of Health Outcomes and Behavior, Moffitt Cancer Center, Tampa, FL; ‡ Department of Pediatrics, The University of Texas MD Anderson Cancer Center, Houston, TX; § Department of Surgical Oncology, The University of Texas MD Anderson Cancer Center, Houston, TX.

We thank Ghielmini and Zingg^1^ for their constructive review of our 2023 publication titled, “Effects of a Pragmatic Home-based Exercise Program Concurrent With Neoadjuvant Therapy on Physical Function of Patients With Pancreatic Cancer: The PancFit Randomized Clinical Trial”.^2^

In the PancFit clinical trial, we randomized 151 patients with resectable/borderline resectable pancreatic cancer to enhanced usual care versus a home-based, multimodal exercise program administered concurrently with neoadjuvant chemotherapy before surgical resection. The primary outcome was submaximal exercise capacity as measured by the 6-minute walk test distance (6MWD). Our hypothesis was that preoperative exercise would improve physical function before pancreatectomy; the trial was designed to detect a difference of 30 m in 6MWD change before surgery between the 2 arms. There was no significant difference in 6MWD change between study arms, as presented in Table 3. Thus, we cannot, and did not, conclude that the randomization to the prescribed prehabilitation exercise program resulted in better physical functioning or clinical outcomes.

Why did this home-based, unsupervised exercise program elicit no significant improvements in submaximal exercise capacity or other physical functioning and clinical outcomes compared to the enhanced usual care condition? We acknowledge and appreciate the critique from Ghielmini and Zingg that our published abstract and discussion section lacked explicit rejection of the a priori hypothesis guiding our trial, but we found it important to focus the article on potential answers to this question. To this end, we observed and highlighted that participants in both study conditions engaged in notable physical activity and self-reported exercise, between which the only statistically significant difference was an increase in muscle-strengthening exercise in the intervention group. We discussed that the enhanced usual care condition in this study, in which participants were encouraged to be physically active by clinical teams and research personnel, seemed to be similarly effective in encouraging physical activity. Indeed, the Hawthorne effect (study participants knew they were being monitored) and the use of activity trackers may have influenced exercise behavior in the short term.^3–5^ Therefore, we found it important to report associations among physical activity and exercise variables and changes in study outcomes in pooled analyses (Table 5). As these analyses demonstrated, there were apparent fitness, physical functioning, self-reported, and clinical benefits to physical activity and exercise during the study period, regardless of study group allocation. We are confident in the clinical relevance of these findings and are actively using them to develop more effective interventional strategies and a stronger trial design in this context.

We applaud the accurate and important insight that Ghielmini and Zingg have brought forward and, as they noted, we appreciate that *Annals of Surgery Open* is committed to publishing clinically relevant results from a null trial. We hope that our trial results and the resulting commentary do not suggest the absence of benefits from preoperative exercise for patients with pancreatic cancer. It is important to develop and evaluate strategies to increase physical activity in this and other cancer-specific settings.

## ACKNOWLEDGMENT

All four authors included here contributed to the design, implementation, and analysis of the original clinical trial. They all contributed to writing of this Letter to the Editor.
